# Mimics and Diagnostic Pitfalls of Anti‐Adenylate Kinase 5 Limbic Encephalitis

**DOI:** 10.1002/cns.70757

**Published:** 2026-01-22

**Authors:** Jierui Wang, Tong Yi, Guoyu Wang, Minjin Wang, Dong Zhou, Jinmei Li

**Affiliations:** ^1^ Department of Neurology West China Hospital of Sichuan University Chengdu China; ^2^ Department of Laboratory Medicine West China Hospital of Sichuan University Chengdu China; ^3^ Department of Laboratory Medicine The First People's Hospital of Longquanyi District Chengdu China

**Keywords:** adenylate kinase 5 antibody, autoimmune encephalitis, limbic encephalitis, mimics of autoimmune encephalitis, overdiagnosis

## Abstract

**Background:**

The clinical understanding of limbic encephalitis associated with antibodies against adenylate kinase 5 (AK5) remains limited. Misinterpretation of antibody test results may lead to diagnostic errors and inappropriate management. We aim to assess the frequency of anti‐AK5 encephalitis overdiagnosis and identify common diagnostic pitfalls.

**Methods:**

Cases of confirmed and mimicking anti‐AK5 limbic encephalitis from January 2021 to July 2024 using established criteria for autoimmune encephalitis (AE) were reviewed. AK5 mimics were defined as patients initially suspected of AE with a positive AK5 autoantibody result, but who ultimately received an alternative final diagnosis.

**Results:**

A total of 21 patients were included (57.1% female; median age 34 years; range 14–82). Only 3 patients (14%) were diagnosed with definite anti‐AK5 limbic encephalitis, while 18 patients (86%) were classified as AK5 mimics. Serum autoantibodies were predominantly of the IgG3 subclass, with titers ranging from 1:10 to 1:100. The mimics included primary psychiatric disorders (22%), central nervous system (CNS) infections (22%), other inflammatory disorders (28%), epilepsy (16%), neurodegenerative diseases (6%) and metabolic encephalopathy (6%). The most frequent confounding factor in misdiagnosis was the presence of prominent psychiatric and behavioral symptoms, seen in 50% (9 of 18) of AK5 mimics. The second most common confounder was the presence of low serum antibody titers or isolated serum positivity without corresponding cerebrospinal fluid (CSF) findings (< 1:100), observed in 94% (17 of 18) of mimics.

**Conclusion:**

Mimics of anti‐AK5 encephalitis are common and that misdiagnosis is often driven by non‐specific symptoms and clinically irrelevant antibody results.

## Graphical abstract

Mimics of anti‐AK5 encephalitis are common and that misdiagnosis is often driven by non‐specific symptoms and clinically irrelevant antibody results. The absence of cognitive impairment as a major symptom, prominence of psychiatric and behavioral symptoms, isolated from serum positivity or low CSF antibody titers (< 1:100), and objective clinical improvement following immunotherapy should prompt reconsideration of the diagnosis. The figure was drawn by Figdraw.

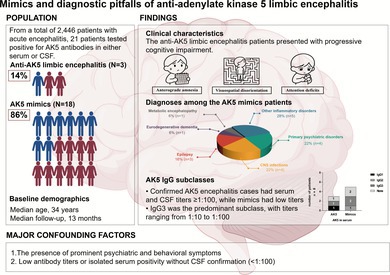

## 1 Introduction

Limbic encephalitis associated with antibodies against adenylate kinase 5 (AK5) is a rare, non‐paraneoplastic form of autoimmune encephalitis and remains poorly characterized due to its rarity [1]. Reported cases, primarily from North America and Europe, suggest that anti‐AK5 encephalitis typically presents with subacute anterograde amnesia, often without seizures, and may be preceded by a prodromal phase of asthenia or mood disturbances [2, 3]. Immunologically, anti‐AK5 antibodies target intracellular antigens, which have been associated with a poor response to immunotherapy, similar to the clinical course of paraneoplastic neurological syndromes [4].

Acute diagnosis of autoimmune encephalitis (AE) often hinges on the detection of neuronal autoantibodies in serum and cerebrospinal fluid (CSF). However, the clinically irrelevant antibody findings may lead to misdiagnosis, inappropriate treatment, and delays in identifying alternative, often treatable conditions. Given the potential for serious side effects associated with immunotherapy and the broad differential diagnosis for AE, improving diagnostic specificity for anti‐AK5 encephalitis is essential for clinical practice [5]. This study aims to determine the prevalence of anti‐AK5 encephalitis misdiagnosis through retrospective case analysis, and to identify commonly encountered mimics and diagnostic red flags that may help clinicians avoid misdiagnosis.

## 2 Methods

### 2.1 Patient Selection and Clinical Information

**FIGURE 1:**
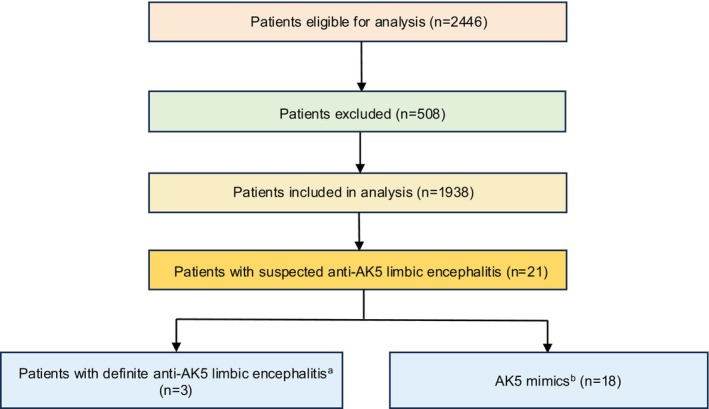
A flowchart of the study subjects. Patients referred to or diagnosed with anti‐AK5 limbic encephalitis at West China Hospital, Sichuan University. ^a^Confirmed anti‐AK5 limbic encephalitis: patients referred with a diagnosis of anti‐AK5 limbic encephalitis that was confirmed at the West China Hospital, Sichuan University. ^b^AK5 mimics: Patients referred with a diagnosis of anti‐AK5 limbic encephalitis that was revised to another diagnosis at the West China Hospital, Sichuan University.

Between January 2021 and July 2024, 2446 patients with acute encephalitis were enrolled at West China Hospital of Sichuan University, Chengdu Shang Jin Nan Fu Hospital, and West China Tianfu Hospital. Among them, 1938 patients underwent AE and paraneoplastic antibody testing using both serum and CSF samples, prior to the initiation of immunotherapy or any other treatment (Figure 1). From this cohort, 21 patients tested positive for AK5 antibodies in either serum or CSF and were included in this study.

Collected clinical data included age at onset, sex, previous comorbidities, clinical symptoms, laboratory parameters, brain MRI findings, electroencephalogram (EEG) findings, time from symptom onset to hospital admission, timing and type of immunotherapy, treatment modalities, and length of hospital stay that were extracted from electronic medical records. The modified Rankin Scale (mRS) scores were used to assess disease severity.

This study was approved by the Ethics Committee of West China Hospital, Sichuan University (permit number: 2021‐1529). Informed consent was obtained from all patients or their legal representatives.

### 2.2 Diagnostic Criteria and Definitions

At least two trained neurologists or psychiatrists independently reviewed all clinical data to establish final diagnoses. All patients were evaluated according to the 2016 AE criteria [6]. Definite anti‐AK5 limbic encephalitis was defined as the presence of AK5 antibodies in serum or CSF along with a compatible clinical syndrome. AK5 mimics were defined as patients with an initial high clinical suspicion for AE and a positive AK5 antibody result, but who were ultimately diagnosed with an alternative condition [5]. Patients exclusively demonstrating pleocytosis and altered mental status, new onset seizures, or focal deficits without other specific evidence of an infectious or inflammatory cause were labeled as other inflammatory disorders [5]. The diagnostic classification of epilepsy was based on the International League Against Epilepsy (ILAE) criteria [7]. Neurodegenerative dementia patients fulfilled the core clinical criteria for dementia, as defined by the National Institutes of Aging‐Alzheimer Association workgroups [8]. Diagnoses of primary psychiatric disorders were established according to the Diagnostic and Statistical Manual of Mental Disorders, Fifth Edition.

### 2.3 Antibody Detection

The CSF and serum samples were tested using commercially available indirect immunofluorescence Cell‐based assays (CBA) (EUROIMMUN, Lübeck, Germany) for the detection of following autoantibodies: NDMAR, LGI‐1, AMPAR, CASPR2, GABAbR, mGluR1, mGluR5, AK5, NCDN, DPPX, and IgLON5. The samples were considered positive when the immunofluorescence was observed at the titers greater than 1:10. The antibody titers were measured using serial dilutions of serum and CSF until the reactivity was no longer visible. For analytical purposes, we classified antibody titers as follows: low titer (≤ 1:100) and high titer (> 1:100). Dot blot assays were used to detect antibodies against neuronal intracellular antigens, including Hu, Yo, Ri, CV2, Ma1, Ma2, Amphiphysin, Titin, PKCγ, GAD65, ZIC4, SOX1, DNER/Tr, Homer 3, and recoverin.

### 2.4 Indirect Immunofluorescence (IIF) Assay

Serum and CSF samples were evaluated using a cryosectioned (4 μm) section of adult mouse or monkey tissues, including the cerebellum, midbrain, cerebral cortex, and hippocampus [9, 10, 11]. A 10% PBST solution was prepared by mixing phosphate‐buffered saline (PBS) with Triton X‐100. Samples were centrifuged at 800 *g* for 5 min, and the supernatant was diluted with PBST (serum at a 1:10 dilution, while the CSF remained undiluted). The prepared samples were applied to tissue sections and incubated at room temperature for 1 h, followed by 3 to 5 washes with PBST. Fluorescein (FITC) AffiniPure Goat Anti‐Human IgG, Fcγ fragment specific secondary antibody (Jackson ImmunoResearch Inc., PA, USA) was diluted 1:200 in PBST and used as the secondary antibody. Sections were incubated for 30 min and washed again. Fluorescent signals were visualized using a fluorescence microscope.

### 2.5 Western Blotting Assay

Results from the Indirect Immunofluorescence Assay (IIF) indicating the presence of AK5 IgGs were validated through Western blot analysis utilizing recombinant full‐length human AK5 protein (UNIPROT ID: Q9Y6K8). Following sodium dodecyl sulfate–polyacrylamide gel electrophoresis (SDS‐PAGE), proteins were transferred to a nitrocellulose membrane. Patient samples were diluted 1:30 in blocking buffer (5% milk in 1× PBST) applied to the membrane, and incubated for 15 min, followed by PBST washes. The membrane was then incubated with Horseradish Peroxidase AffiniPure Goat Anti‐Human IgG (1: 5000; Jackson ImmunoResearch Inc., PA, USA) for 30 min at room temperature. The immunoreactive bands were developed using SuperSignal West Pico PLUS (Thermo Fisher Scientific Inc., CA, USA) following the manufacturer's instructions. The protein bands corresponding to AK5 were compared to a positive control. The results were reported qualitatively as either positive or negative.

### 2.6 Detection of AK5 Antibodies

Anti‐AK5 IgG subclass analysis was conducted using a standardized protocol previously described in the literature [12]. Serum samples from seropositive patients on teased were diluted 1:10 in working buffer and incubated for 1 h with fixed HEK293T cells expressing AK5 (EUROIMMUN, Lübeck, Germany). For subclass analysis, we used Fluorescein (FITC)‐conjugated secondary antibodies diluted 1:200, namely F0767 (Anti‐Human IgG1‐FITC Antibody), F4516 (Anti‐Human IgG2–FITC antibody), F4641 (Anti‐Human IgG3–FITC antibody), F9890 (Anti‐Human IgG4–FITC Antibody), IgM (Anti‐Human IgM–FITC antibody), and IgA (Anti‐Human IgA–FITC antibody), all purchased from Merck, Germany.

### 2.7 Statistical Analysis

Quantitative data were presented as medians with interquartile ranges (IQRs; 25% to 75%), while categorical data were expressed as percentages or ratios. Statistical analyses were performed using GraphPad Prism 8.0 and SPSS 22.0. Comparisons between categorical variables were made using the Chi‐square (*χ* ^2^) test. Continuous variables were compared using the Mann–Whitney *U* test. Correlation between continuous variables was evaluated using Spearman rank correlation and Pearson correlation coefficients, as appropriate. A two‐sided *p*‐value < 0.05 was considered statistically significant.

## 3 Results

### 3.1 Patient Characteristics

We retrospectively analyzed clinical and paraclinical data from 21 patients with suspected anti‐AK5 limbic encephalitis. The median follow‐up was 13 months (interquartile range [IQR] 10–19, range 8–40). The median age at disease onset was 34 years (IQR 16–67), with 42.9% of patients being male. Of these, three patients (14.3%) were diagnosed with definite anti‐AK5 limbic encephalitis, while the remaining 18 (85.7%) were diagnosed with AK5‐mimics. There were no significant differences in median age or sex distribution between the two groups. One patient with definite anti‐AK5 limbic encephalitis had extranodal marginal zone lymphoma of mucosa‐associated lymphoid tissue. Clinical and laboratory features of the anti‐AK5 limbic encephalitis mimics are summarized in Table 1, along with comparisons to the anti‐AK5 limbic encephalitis group.

**TABLE 1:** Baseline demographics, clinical and laboratory data in patients with confirmed diagnosis and mimics of Anti‐AK5 Limbic Encephalitis.

	Accurately diagnosed	Mimics	*p*
Number of subjects	3	18	
Demographic characteristics			
Female (%)	2 (66.7%)	10 (55.6%)	0.79^a^
Age at first symptom onset, median (IQR), years	68 (53, 82)	32 (16, 57)	0.06^b^
Time from first symptom to hospital, median (IQR), weeks	2 (2, 36)	0.5 (0.3, 4.5)	0.14^b^
Baseline mRS score, median (IQR)	3	4	0.58^a^
1 (*n*, %)	0 (0%)	0 (0%)	
2 (*n*, %)	0 (0%)	0 (0%)	
3 (*n*, %)	2 (66.7%)	14 (77.8%)	
4 (*n*, %)	1 (33.3%)	2 (11.1%)	
5 (*n*, %)	0 (0%)	2 (11.1%)	
Clinical features			
Cognitive disorders	3 (100%)	3 (16.7%)	**0.02** ^a^
Psychiatric‐behavior symptoms	2 (66.7%)	9 (50%)	0.93^a^
Seizures	1 (33.3%)	4 (22.2%)	0.75^a^
Dystaxia	0 (0%)	2 (11.1%)	0.65^a^
Disturbance of consciousness	0 (0%)	1 (5.6%)	0.29^a^
Sleep disorders	1 (33.3%)	2 (11.1%)	0.90^a^
≥ 3 symptoms^c^, *n* (%)	3 (100%)	4 (22.2%)	**0.02** ^a^
Tumor	1 (33.3%)	0 (0%)	0.14^a^
Diagnostic features			
Brain MRI, abnormality, *n* (%)	2 (66.7%)	6 (33.3%)	0.65^a^
EEG, abnormality, *n* (%)	0 (0%)	2/6 (33.3%)	—
CSF, abnormality, *n* (%)			
Leukocyte counts > 5 cells/μL	1 (33.3%)	3 (16.7%)	0.91^a^
Protein > 0.45 g/L	1 (33.3%)	4 (22.2%)	0.97^a^
Anti‐AK5 Antibody Status in CSF (%)			
Titer ≥ 1:100	3 (100%)	1 (5.6%)	**0.003** ^a^
Titer < 1:100 or negative	0 (0%)	17 (94.6%)	**0.002** ^a^
Anti‐AK5 Antibody Status in Serum (%)			
Titer ≥ 1:100	3 (100%)	2 (11.1%)	**0.01** ^a^
Titer < 1:100	0 (0%)	15 (83.3%)	**0.01** ^a^
Other Neural Autoantibodies Positive (%)	0 (0%)	5 (27.8%)	0.75^a^
Treatment modalities, *n* (%)			
None	1 (33.3%)	7 (38.9%)	0.65^a^
Immunotherapies			
IVIg alone	1 (33.3%)	5 (27.8%)	0.62^a^
IVMP alone	1 (33.3%)	2 (11.1%)	0.90^a^
IVIg combined with IVMP	0 (0%)	4 (6.9%)	0.91^a^
Hospital outcomes			
Length of hospital stay, median (IQR), days	12 (11, 12)	13 (11, 23)	0.2^b^
Stabilized, *n* (%)	1 (33.3%)	13 (72.2%)	**0.019** ^a^
Improvement, *n* (%)	0 (0%)	4 (6.9%)	0.91^a^
Poor response to treatment, *n* (%)	2 (66.7%)	1 (5.6%)	**0.04** ^a^

*Note:* Bold entries indicate *p* < 0.05.

Abbreviations: AK5, anti‐adenylate kinase 5; CSF, cerebrospinal fluid; EEG, Electroencephalogram; IQR, interquartile range; IVIg, intravenous immunoglobulin; IVMP, intravenous Methylprednisolone; MRI, magnetic resonance imaging; mRS, Modified Rankin Scale.

^a^ Fisher's exact test.

^b^ Mann–Whitney *U* test.

^c^ Five most frequent symptoms in AE: working memory problems, new‐onset seizures, behavioral disorders, sleeping disorders, and psychiatric symptoms.

Cognitive disorders were notably more common in patients with anti‐AK5 limbic encephalitis. All three cases (100%) presented with severe, progressive cognitive impairment, including anterograde amnesia, visuospatial disorientation, and attention deficits at disease onset (Table 2). One patient eventually developed dementia. In addition, anti‐AK5 limbic encephalitis patients more frequently exhibited ≥ 3 of the following symptoms compared to mimics (100% vs. 22.2%, *p* < 0.05): working memory deficits, new‐onset seizures, behavioral disorders, psychiatric symptoms, and sleep disorders. Among 3 confirmed AK5 encephalitis cases, all had serum and CSF titers ≥ 1:100, while the 18 mimic cases showed predominantly low titers: 17/18 (94.4%) had CSF < 1:100 or negative, 15/18 (83.3%) serum < 1:100. High titers (≥ 1:100) were exclusive to patients with definite anti‐AK5 limbic encephalitis, indicating low titers correlate with AK5 mimics. Immunotherapy was administered to nearly half of the patients in both groups. While 13 of 18 mimic cases achieved clinical remission or stabilization, none of the patients with confirmed anti‐AK5 limbic encephalitis showed clear improvement following immunotherapy.

**TABLE 2:** Clinical characteristics, ancillary tests and treatment of the 3 patients with anti‐AK5 antibodies.

Patient sex, age (years)	Main clinical features	Brain MRI	Epileptic abnormalities on EEG	CSF	Therapy	Preferred sample types; detection methodology	Short‐term outcome
1 F, 82	Amnesia, depression, altered circadian rhythm, psychomotor agitation, visual hallucinations	Left temporal lode T2/FLAIR signal↑	No	1 cell/mL, proteins 0.48 g/L, OCB NA	None	Serum and CSF; TBA (IIF) and CBA	Mild improvement
2 F, 53	Amnesia, followed by focal seizures with impaired awareness	Global T2/FLAIR signal↑	Spikes, spike‐and‐waves T4–T6	5 cell/mL, proteins g/L, OCB NA	Steroids; cyclophosphamide	Serum and CSF; TBA (IIF) and CBA	Poor response to treatment
3 M, 68	Amnesia, depression, anxiety	Bihippocampal, frontal and parietal atrophy	No	2 cell/mL, proteins 0.36 g/L, OCB NA	IVIG	Serum and CSF; TBA (IIF) and CBA	Poor response to treatment

Abbreviations: AK5, anti‐adenylate kinase 5; CBA, cell‐based assay; CSF, cerebrospinal fluid; F, female; FLAIR, fluid‐attenuated inversion recovery; IVIg, intravenous immunoglobulin; M, male; MRI, magnetic resonance imaging; NA, not available; OCB, oligloclonal band; TBA, tissue‐based assay.

### 3.2 AK5 Mimics and Confounding Factors

**FIGURE 2:**
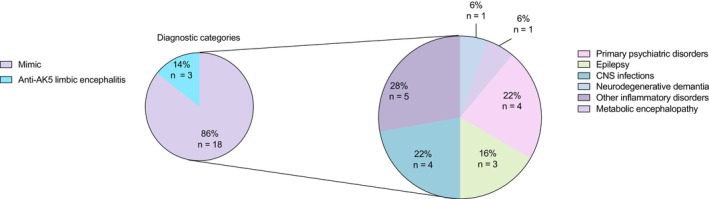
Overview of Diagnostic Categories Specified (Total Group vs. Mimics). The pie charts on the left represent the specific inflammatory categories (depicted in blue) and AK5 mimics (depicted in purple) in the total group (*n* = 21). Diagnostic subcategories of AK5 mimics are demonstrated in the category on the right side.

In 18 of the 21 anti‐AK5 antibody‐positive patients (85.7%), the final diagnosis was revised to an alternative neurological disorder (Figure 2). Diagnoses among the mimic group included primary psychiatric disorders (22%), CNS infections (22%), other inflammatory disorders (28%), epilepsy (16%), neurodegenerative dementia (6%), and metabolic encephalopathy (6%).

The most frequent clinical confounding factor leading to misdiagnosis was the presence of psychiatric and behavioral symptoms, observed in 9 of 18 (50%) AK5 mimics patients. These included agitation, aggression, depression, anxiety, and psychosis with delusions or visual hallucinations (Table 1). The second most common confounder was the detection of anti‐AK5 antibodies solely in serum or at low titers in CSF (< 1:100), which was observed in 17 of 18 (94%) mimic cases.

### 3.3 AK5 IgG Autoantibodies in Anti‐AK5 Limbic Encephalitis and Mimics

**FIGURE 3:**
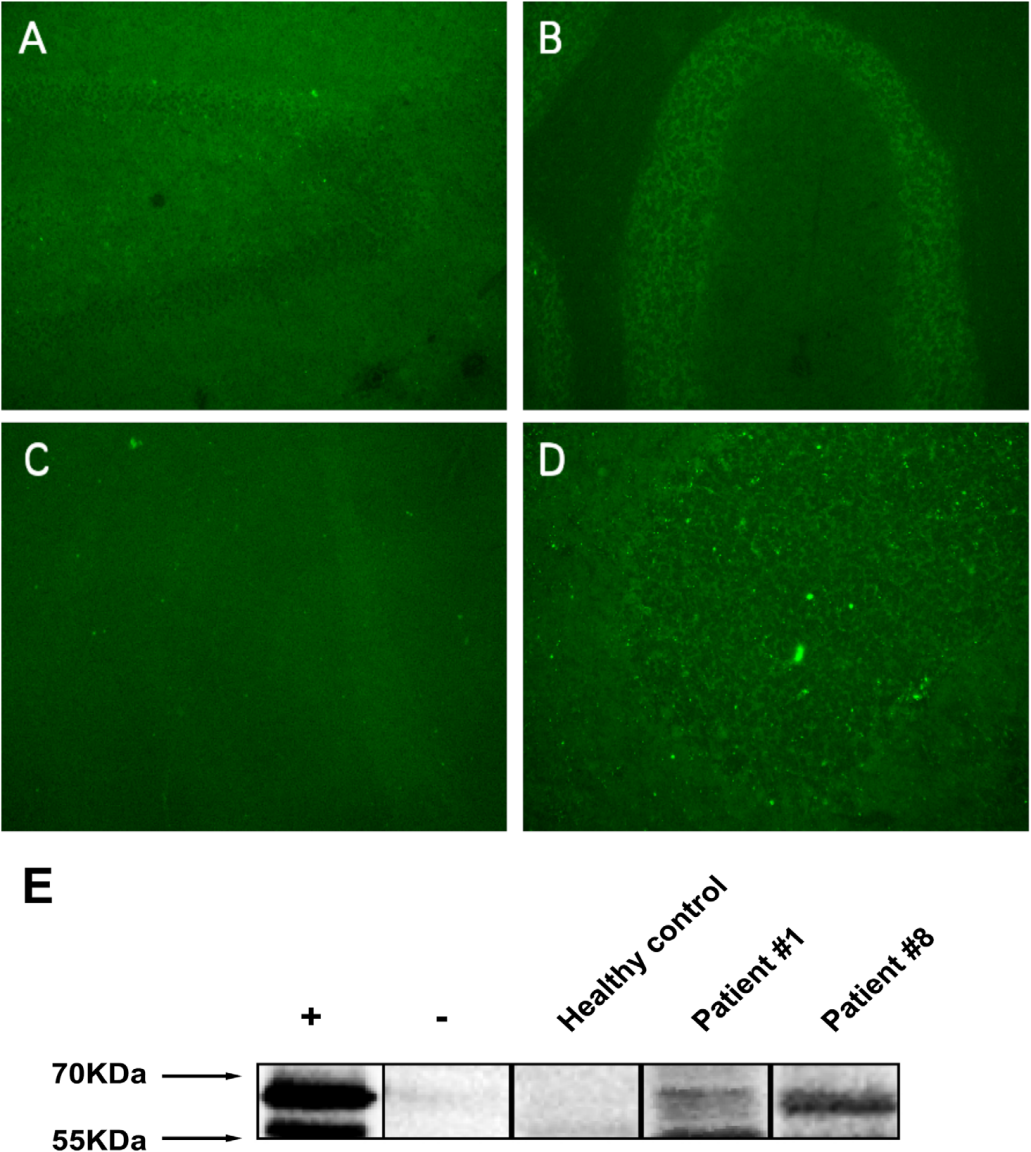
Indirect immunofluorescence on mouse brain shows binding of IgG derived from serum of AK5‐IgG positive patients in a diffuse pattern in the hippocampus (A) and cerebellum (B). Antibody binding was seen in the cerebellum (C, higher magnification D) with cytoplasmic staining of the Purkinje cells (arrow) in monkey brain. (E) Western blot of sera on HEK293T cell lysate transfected with full length AK5 (+) and untransfected control HEK293T cell lysate (−). Representative image of patient #1 (Definite AK5 encephalitis) and patient #8 (AK5 mimics) showing AK5 band, while healthy control shows no binding.

According to the literature, a positive test should be confirmed by at least two 2 techniques such as brain immunohistochemistry and CBA to verify presence of antibodies [13]. Given that most AK5 mimics tested negative for AK5 antibodies in the CSF, we further examined the epitope distribution and staining patterns of serum AK5 IgG. Using IIF on mouse and monkey brain sections, both definite AK5 encephalitis patients and AK5 mimic patients displayed a similar IgG staining pattern to that reported in previous literature, characterized by diffuse cytoplasmic binding in hippocampal granular and pyramidal neurons, and Purkinje cells in the cerebellum (Figure 3A–D) [4]. Western blot analysis using recombinant full‐length human AK5 protein (UNIPROT ID: Q9Y6K8) confirmed IIF AK5 positivity and supported IIF findings (Figures 3E).

### 3.4 AK5 IgG Subclasses in Anti‐AK5 Limbic Encephalitis and Mimics

**FIGURE 4:**
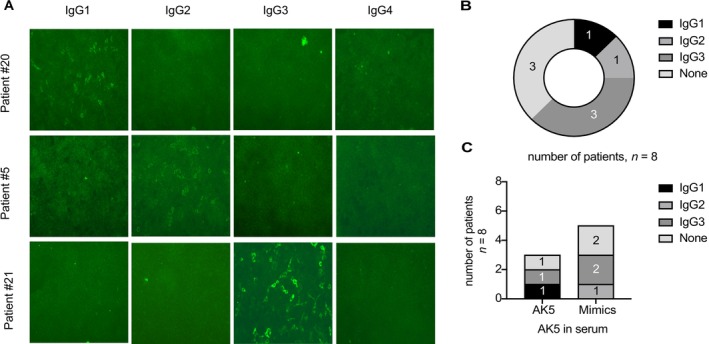
IgG subclass determination of AK5 IgG‐positive sera. (A) IgG subclass determination via indirect immunofluorescence of AK5‐transfected HEK293T cells. Representative pictures displaying reactivity against IgG1 only (patient #20), IgG2 only (#5), IgG3 only (#21). No sample reacted against IgG4. Patient #20 and #5 were definite AK5 encephalitis patients, and patient #21 was classified as an AK5 mimic. (B, C) Distribution of IgG subclasses for serum samples.

To explore the potential pathogenic contribution of IgG subclasses, we analyzed serum from eight patients (three with definite AK5 encephalitis and five with mimics). The representative staining patterns shown in Figure 4A illustrate the range of IgG subclass reactivities observed in our cohort, and no association was found between any IgG subclass and clinical diagnosis or symptomatology.

Across all patients (both definite AK5 encephalitis and mimics), IgG3 was the predominant subclass, with titers ranging from 1:10 to 1:100. One patient tested positive for IgG1 (12.5%), one patient was positive for IgG2 (12.5%), while three patients were positive for IgG3 (37.5%). The remaining three samples showed no detectable IgG subclass reactivity. No IgG4, IgG A, or IgG M antibodies were identified in any serum sample.

## 4 Discussion

In this retrospective cohort study, we identified common anti‐AK5 encephalitis mimics and highlighted key red flags to prevent misdiagnosis. Our findings show that anti‐AK5 encephalitis mimics are diverse and significantly outnumber confirmed anti‐AK5 encephalitis case diagnoses, with misdiagnosis occurring approximately six times more frequently. The most common alternative diagnoses included primary psychiatric disorders, CNS infections, other inflammatory disorders, epilepsy, neurodegenerative dementia, and metabolic encephalopathies.

Anti‐AK5 encephalitis is a rare but severe autoimmune limbic encephalitis. To date, only 31 cases have been reported, and common diseases often account for a high proportion of cases mistaken for anti‐AK5 encephalitis [14]. While cognitive impairment and psychiatric‐behavioral symptoms have been emphasized as diagnostic clues for anti‐AK5 encephalitis [15], our study demonstrates that psychiatric and behavioral features are also the most frequent sources of diagnostic error. These symptoms were present in 50% of patients ultimately classified as mimics, underscoring the need for caution and thorough evaluation. Given the rarity of anti‐AK5 encephalitis, other more prevalent conditions, particularly primary psychiatric disorders, CNS infections, more common autoimmune encephalitis, and neurodegenerative diseases, should be carefully ruled out in the differential diagnosis. In addition to typical symptoms, our study also identified rare symptoms such as seizures and sleep disturbances. Prior reports have documented additional atypical features including altered consciousness disturbances and taste disorders [16, 17]. However, our understanding of the clinical spectrum of anti‐AK5 encephalitis remains incomplete, particularly in East Asian populations, where few large cohort studies exist. Patients with atypical clinical phenotypes pose significant diagnostic challenges and are at higher risk of being either misdiagnosed with or overlooked for anti‐AK5 encephalitis [15]. These findings emphasize the importance of heightened diagnostic vigilance and careful clinical correlation, especially in ambiguous cases [13, 18]. Notably, more than half of the patients misdiagnosed as having anti‐AK5 encephalitis received immunotherapy. This finding reinforces the critical need for accurate early differentiation, as inappropriate immunotherapy, such as high‐dose corticoids, can lead to adverse outcomes, including symptom worsening in psychiatric disorders or exacerbation of CNS infections [13, 19]. Beyond clinical presentation, we identified several red flags that may aid in distinguishing anti‐AK5 encephalitis from its mimics. These include the absence of prominent cognitive impairment, predominant psychiatric symptoms, antibody detection limited to serum with low CSF titers, and clinical improvement following immunotherapy, which should prompt reevaluation for alternative diagnoses (Table 3). Identifying these warning signs can guide early and appropriate treatment, while minimizing the risk of unnecessary and potentially harmful immunotherapies in patients without true autoimmune pathology.

**TABLE 3:** Red flags to consider mimics of Anti‐AK5 Limbic Encephalitis.

**Presentation**
Absence of cognitive disorders
Psychiatric‐behavior symptoms are absent, or not a major symptom
Seizure is the predominant feature
**Ancillary testing**
Lower titer or absence of Anti‐AK5 antibodies in CSF
**Clinical course**
Objective improvement with immunotherapy

Abbreviations: AK5, anti‐adenylate kinase 5; CSF, cerebrospinal fluid.

The second most common confounding factor was clinically irrelevant antibody test results, observed in 94% of mimics. This likely reflects both the overuse of AE diagnostic panels and increased clinical awareness. While expanding testing can improve detection, it also increases the likelihood of identifying antibodies in patients without supportive clinical evidence. This underscores the importance of applying diagnostic tools judiciously and adhering strictly to established clinical criteria. Interestingly, most AK5 encephalitis mimics in our cohort met the 2016 clinical criteria for possible autoimmune encephalitis by Graus et al. [6]. However, as noted by others, this category lacks specificity and should not be considered definitive without corroborative findings [5, 20]. There is a clear need for better characterization of rare AE syndromes, both in terms of pathogenesis and clinical phenotype. Currently, antibody positivity remains a key diagnostic criterion for AE [21], but interpreting rare antibody findings, such as AK5‐IgG, requires consistency between clinical features and antibody profiles to avoid overinterpretation. In our study, AK5‐antibody presence in both true and mimic cases suggests that serum positivity can be nonspecific and not necessarily the primary driver of the current neurological disease. AK5‐antibody might be occurring as a bystander effect in other brain disorders, or result from the production of cross‐reactive IgG, or represent an epiphenomenon of neuronal injury or a remnant of a prior immune event, rather than a direct cause of pathology. Our data suggest that high‐titer AK5‐antibody titers (≥ 1:100) in both serum and CSF may have some predictive value in identifying neurological autoimmunity in clinically suspected AE cases [22]. Consistent with previous studies, our findings support the routine analysis of paired CSF and serum samples in suspected AE [23]. Therefore, our findings endorse the principle that antibody detection provides supportive, but not definitive, evidence of autoimmune pathogenesis.

This study has several limitations. First, as a retrospective study, there is potential for selection bias, which means that the reported associations should be interpreted with caution. Second, our study is limited by its retrospective design and the lack of seizure frequency monitoring and neuropsychological assessments. Third, we relied on the mRS score as a short‐term outcome measurement, which may not fully capture cognitive, behavioral, and psychosocial outcomes. Consequently, outcomes directly linked to the disease, such as persistent cognitive deficits, psychiatric and behavioral symptoms, or risk of seizure recurrence, may have been underreported. Future studies should aim for larger sample sizes and incorporate more comprehensive outcome assessments to better evaluate long‐term efficacy and prognosis [24]. Fourth, the distinction between pathogenic and non‐pathogenic AK5 antibodies was based primarily on clinical follow‐up and integration of neurological signs. IIF and WB confirmed antibody presence in both true and mimic cases, but did not help in discrimination. Finally, we did not use live‐CBA for AK5 detection due to laboratory constraints, which may influence the accuracy of our findings. We will use a broader panel of advanced detection methods to improve the specificity and reliability of AK5 antibody identification in a relatively ideal environment.

## 5 Conclusions

In conclusion, our study demonstrates that mimics of anti‐AK5 encephalitis are common, and misdiagnosis remains a significant challenge. We identified key clinical and preclinical features that can aid in distinguishing anti‐AK5 encephalitis from mimics. The absence of cognitive impairment as a major symptom, prominence of psychiatric and behavioral symptoms, isolated from serum positivity or low CSF antibody titers (< 1:100), and objective clinical improvement following immunotherapy should prompt reconsideration of the diagnosis. Recognizing these red flags can support more accurate diagnoses, guide appropriate treatment, and reduce the risk of unnecessary and potentially harmful immunotherapy in patients without autoimmune encephalitis.

## Author Contributions

J.L. conceived and designed the study and supervised the study. J.W. contributed to the implementation of the study protocol, the statistical analysis, and the writing of the paper. J.L. and D.Z. contributed to the critical revision of the paper. J.W., T.Y., G.W., M.W., and J.L. collected and interpreted the data. All authors had full access to all the data in the study and accepted responsibility to submit for publication. All authors revised the manuscript and approved the final manuscript as submitted.

## Funding

This study was supported by the China Postdoctoral Science Foundation (2024M762242).

## Ethics Statement

The protocol was approved by the Ethics Committee of West China Hospital of Sichuan University (permit number: 2021‐1529). Informed consent was obtained from the patients or their families.

## Conflicts of Interest

The authors declare no conflicts of interest.

## Data Availability

The data that support the findings of this study are available on request from the corresponding author. The data are not publicly available due to privacy or ethical restrictions.
